# Neuromuscular Strategies in Stretch–Shortening Exercises with Increasing Drop Heights: The Role of Muscle Coactivation in Leg Stiffness and Power Propulsion

**DOI:** 10.3390/ijerph17228647

**Published:** 2020-11-21

**Authors:** Riccardo Di Giminiani, Aldo Giovannelli, Lorenzo Capuano, Pascal Izzicupo, Andrea Di Blasio, Francesco Masedu

**Affiliations:** 1 Department of Biotechnological and Applied Clinical Sciences, University of L’Aquila, 67100 L’Aquila, Italy; aldo.giovannelli@univaq.it (A.G.); lorenzo.capuano88@libero.it (L.C.); francesco.masedu@univaq.it (F.M.); 2 Department of Medicine and Aging Sciences, University “G. D’Annunzio” of Chieti-Pescara, 66100 Chieti, Italy; izzicupo@unich.it (P.I.); andrea.diblasio@unich.it (A.D.B.)

**Keywords:** drop jump, momentum, pre-activation, co-contraction, EMG activity

## Abstract

When applying drop jump exercises, knowing the magnitude of the stimulus is fundamental to stabilize the leg joints and to generate movements with the highest power. The effects of different drop heights on leg muscles coactivation, leg stiffness and power propulsion were investigated in fifteen sport science students. Drop jumps from heights of 20, 30, 40, 50, and 60 cm in a random order were performed on a force platform. During each drop jump, the ground reaction force, knee angle displacement, and synchronized surface-electromyography root-mean-square (sEMG_RMS_) activity (vastus lateralis, VL; vastus medialis, VM; rectus femoris, RF; biceps femoris, BF; tibialis anterior, TA and lateral gastrocnemius, LG) were recorded. The coactivation in the pre-contact phase, between VL and BF, VM and BF as well as RF and BF, was dependent on the drop height (*p* < 0.01; effect size (ES) ranged from 0.45 to 0.90). Leg stiffness was dependent on the drop height (*p* < 0.001; ES = 0.27–0.28) and was modulated by the coactivation of VM–BF (*p* = 0.034) and RF–BF (*p* = 0.046) during the braking phase. Power propulsion was also dependent on the drop height (*p* < 0.001; ES = 0.34); however, it was primarily modulated by the coactivation of LG–TA during the braking phase (*p* = 0.002). The coactivation of thigh muscles explains leg stiffness adjustments at different drop heights. On the contrary, the coactivation of shank muscles is mostly responsible for the power propulsion.

## 1. Introduction

Plyometric exercises (i.e., drop jumps) are movements in which the interaction between the human body and the environment is characterized by large transient reaction forces [1]. These exercises involve a muscle action called the stretch–shorten cycle (SSC), where the stretch force is imposed on the neuromuscular system by gravity [2], and when changes in drop height occur, the momentum at impact increases. Therefore, the amount of energy reused if a muscle stretch is followed by a concentric contraction will change [3,4]. The latter biomechanical condition (i.e., the stretch–shorten cycle) strongly stimulates muscular and neurogenic structures [4], even though the more complex and interesting is neurogenic, as the motoneuron activation in the early contact phase is modulated by afferents feedback to increase the impulse through the synchronization of α-motoneurons in the preactivated muscle [5]. The proprioceptive afferents (i.e., primary muscle spindle endings, Golgi tendon organ Ib afferents, cutaneous receptors and mechanoreceptors) are integrated by the CNS, according to the drop height, to control muscle length and tension at impact with the ground and to determine an appropriate leg stiffness regulation that represents a key factor to catapult the elastic energy, stored in the muscle–tendon complex, preactivated and eccentrically stretched to the concentric propulsion phase [5,6,7].

The drop height influences the power output [4,7,8,9,10,11,12,13,14,15], thus modulating feedforward control (pre-programmed) during the pre-contact phase [16,17], which in turn regulates leg stiffness on impact with the ground [18], and feedback control (short, medium and long-latency responses) during the braking and propulsion phases [6,7]. This means that the motor cortical contribution involves the entire drop jump exercise [19] and that a “certain drop height”, relative to the aim of movement (i.e., the power output or height of the jump), determines the integration between feedforward and feedback mechanisms to provide appropriate leg stiffness [19]. Leg stiffness depends on the stiffness of the joints [14,18,20,21], and it can be used as a global mechanical measure of multi-joint coordination to characterize the “stiffness strategy” to control the dynamic interactions between the lower limb and the ground. Leg stiffness can be modulated by varying muscle pre-activation and coactivation before and during foot contact [20].

The functional role of coactivation has been demonstrated for knee joint stability and joint injury protection in isometric contractions [22,23] as well as for preventing ankle sprains in simulated landings [24]. Arai et al. [25] have shown that the coactivation of the muscles around the ankle joint depends on the rebound height but not on the drop height. However, when drop jumps with small angular amplitudes are performed [20,26], the downward displacement of the center of mass during the initial phase of ground contact is decelerated by knee stiffness, which represents the major determinant of leg stiffness [18,20,21]. Therefore, muscle activity, which is dependent on drop height [16,17,27], could be arranged differently between the leg muscles to control leg stiffness and to contribute to power propulsion during drop jumps [20]. Specifically, while leg stiffness is determined functionally by the coactivation of agonist–antagonist leg muscles to ensure joint stability on impact with the ground [18,20,21,22,23], the antigravity muscles, i.e., the plantar flexors and knee and hip extensors [28], are the prime movers during the propulsion phase [29,30,31]. Thus, when the aim is to maximize power propulsion following landing, the neuromuscular strategy involves an alteration in the activation of agonist–antagonist leg muscles; consequently, coactivation will be reduced, and leg stiffness will be adjusted [29,30,31].

From the above studies, we can summarize that selecting an “optimal drop height” is crucial for two reasons: first, it is essential to induce specific physiological adaptations and therefore improving the power performance; second the proper selection of drop height reduces the stress on musculotendinous and bones structures, adjusting leg stiffness. Actually, in the literature the available information regarding the “optimal drop height” to affect the power propulsion and/or the leg stiffness is lacking and it is still unclear if power propulsion and leg stiffness are maximized by the same drop height.

We hypothesized that the drop heights would affect stiffness and power propulsion differently. Specifically, the coactivation of thigh muscles (vastus lateralis, vastus medialis, rectus femoris and biceps femoris) will be dependent on the drop height and it would increase leg stiffness, while the coactivation of the shank muscles (lateral gastrocnemius–tibialis anterior), which are antigravity and propulsive, would have an effect on power propulsion.

The purpose of the present study was to investigate the effect of different drop heights (20, 30, 40, 50, and 60 cm) on the coactivation of leg muscles to understand the neuromechanical strategies used for leg stiffness adjustments and power propulsion during each phase of the jump.

## 2. Materials and Methods

### 2.1. Participants 

Fifteen volunteer sport science students took part in this study (7 males: age 21.8 years (0.14); height 180.3 cm (2.49); body mass 74.6 kg (2.59); body mass index 22.9 kg m^2^ (0.74), and 8 females: age 22.2 years (0.41); height 164.2 cm (2.64); body mass 54.6 kg, (3.54), body mass index 19.8 kg·m^2^ (0.92)). All of the participants had previous experience with drop jump training, and each was involved in physical activities such as gymnastics, swimming, and field activities at least twice a week. The experiments were performed in accordance with the ethical standards of the Helsinki Declaration, and the participants provided written informed consent before taking part in the study. This study was approved by the Internal Review Board (n. 23/2017).

### 2.2. Study Design and Measurements

A single-group repeated-measures study design was used in which the coactivation of leg muscles, ground reaction force, power and leg stiffness were the dependent variables. The independent variable was the drop height (20, 30, 40, 50, and 60 cm).

Power analysis was performed using a non-parametric procedure. We used G-Power (G*Power 3.1.9.4, Heinrich Heine-Dusseldorf University), setting the effect size (according to our previous study) to 0.80 (Cohen’s d-statistics) and alpha equal to 0.05, resulting in a power of 0.80.

The measurements were performed in the biomechanics laboratory at the university. Each participant visited the laboratory on two occasions with a break of at least one day in between visits. During the first lab visit, the participants familiarized themselves with the experimental procedure (subjects repeated each drop height 3–5 times in a random order). During the second lab visit, prior to the sEMG normalization of the selected muscles (vastus lateralis (VL), vastus medialis (VM), rectus femoris (RF), biceps femoris (BF), tibialis anterior (TA) and lateral gastrocnemius (LG)), the participants performed a 15 min warm up (8 min run on a treadmill at a speed of 6 km h^−1^, 2 min dynamic stretching and 5 min mono- and bipedal stance leaps). Each participant then performed drop jumps from different heights on the force platform. The jumps were executed in a random order from the following heights: 20, 30, 40, 50, and 60 cm. Two trials for each drop height were performed, and the jump with the highest flight time was selected for analysis as this value is related to the velocity at take-off and to power propulsion. The pause between trials was 30–40 s. After a 10–12 min rest, the participants performed another set of drop jumps in a random order to assess the reliability of the measurements (Table S1).

#### 2.2.1. Ground Reaction Force

The participants dropped themselves directly on the force platform assuming a standing position on the plyometric box with their hands on the hips. Before dropping on the platform, one leg remained stable in order to secure upright stance, whereas the dominant leg was lifted and projected in front of the body, in this way the participants let themselves fall according to gravity force without jumping slightly high. Participants were asked to touch down on both feet simultaneously and to rebound from the platform with maximal effort and as fast as possible. These verbal instructions (they had previous experience with drop jump training) helped the participants to structure the mental representation [32] of drop jumps with small knee angle flexion (bounce drop jump) at different drop heights [15,26,33,34]. The EMG electrodes were positioned on the dominant side and connected to a data collection unit that was connected to a personal computer via a USB port. Maximum knee flexion was determined using an electrogoniometer placed on the opposite leg. Knee flexion ranged from approximately 122° to 124° on average (an extended knee corresponds to 180°). If knee flexion was more than 5°–7° above the range of the same participant in several trials, the drop jump was repeated.

#### 2.2.2. EMG Activity

EMG activity was recorded using triode electrodes (T3402M, nickel-plated brass, electrode diameter = 1 cm, inter-electrode-distance = 2 cm, Thought Technology Ltd., Montreal, QC, Canada) [35,36]. The electrodes were placed on the dominant side according to the recommendations for the non-invasive electromyographic activity (sEMG) assessment of muscles [37]. Before placing the electrodes, the skin was shaved, abraded with sandpaper and cleaned with alcohol to minimize impedance (<5 kΩ). The electrodes and cables were covered with an elastic band to prevent motion artefacts. The raw EMG signal (Muscle Lab 4000e, Ergotest-Innovation, Porsgrunn, Norway) was amplified and filtered using a preamplifier located near the electrodes to reduce noise from external sources through the signal cables. The EMG preamplifier characteristics were as follows: voltage supply ±5 VDC; input impedance 2 GΩ; common mode rejection rate: 100 dB; input noise level (1 kHz band with): 3u Vcc; output impedance (max.) 10 Ω; output voltage level: ±4 V; gain at 100 Hz: 1000; 3 dB low-cut frequency: 8 Hz; and 3 dB high-cut frequency: 1.2 kHz. The raw EMG signal sampled at 1 kHz was converted to an RMS signal using a hardware circuit network that computed the true root-mean-square level according the standards for reporting the EMG data (International Society of Electrophysiology and Kinesiology, https://isek.org/resources/). The technical data of the RMS conversion circuit are as follows: frequency response (typically) ±3 dB, bandwidth 450 kHz, averaging constant 100 ms, conversion accuracy (total error) ±0.5% of reading. The converted RMS signal was sampled at 100 Hz using a 16 bit A/D converter.

The surface-electromyography root-mean-square (sEMG_RMS_) values were normalized by recording sEMG during maximum isometric contractions [38]. The maximum isometric contractions were performed in leg extension at 45° to record the EMG activity in the VL, VM and RF muscles; in the calf-rise position for LG; during maximal dorsal ankle flexion for TA; and during a leg curl (at a knee angle of 135°) for BF.

### 2.3. Data Analysis

**Figure 1:**
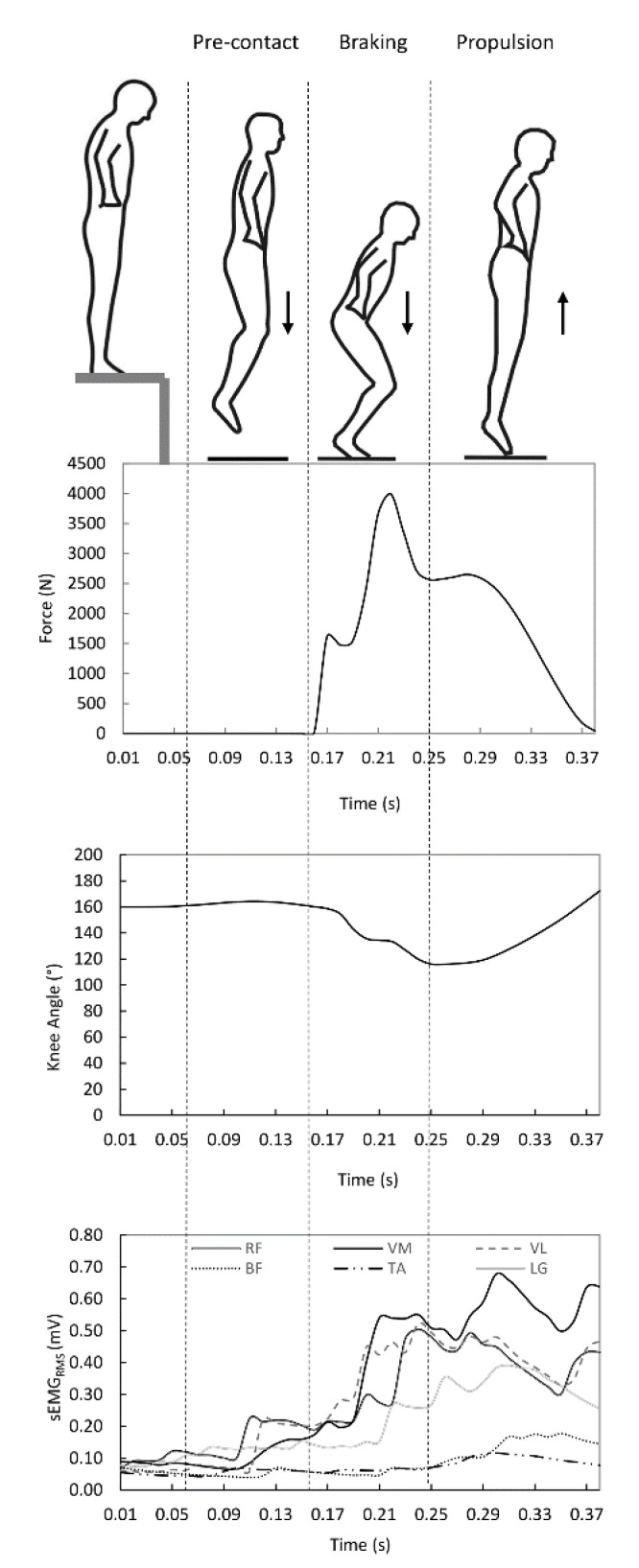
Schematic representation of the time histories of ground reaction force and knee angle displacement during the three phases of the drop jump (pre-contact, braking and propulsion). The surface-electromyography root-mean-square (sEMG_RMS_) of the vastus lateralis (VL), rectus femoris (RF), vastus medialis (VM), biceps femoris (BF), tibialis anterior (TA) and lateral gastrocnemius (LG) muscles was synchronized with the other two variables.

Analysis of the variables was performed in each phase of the drop jump: pre-contact, braking, and propulsion (Figure 1). The pre-contact phase was defined as the 0.10 s period preceding ground contact [3]. Braking and propulsion phases were determined by maximum knee angle displacement on contact with the force platform [12]. All calculations were performed with Sigmaplot software (Systat Software, San Jose, CA, USA). Integrals were calculated using the Simpson method within the math transform option of Sigmaplot. Leg stiffness (*K_leg_*) was calculated as the ratio of the peak vertical ground reaction force (*F_peak_*) and the center of mass displacement (*COM_disp_*) from the initial contact to the time of peak vertical ground reaction force [39]:

(1) $$K_{leg\;=\;\frac{F_{peak}}{COM_{disp}}}$$

COM displacement was calculated by the double integration of the vertical acceleration [40]:

(2) $$COM=\int_{t_{1}}^{t_{2}}vdt$$

#### 2.3.1. Coactivation Index

The coactivation index (CI) was calculated in four different muscle groups (after normalization to the maximal isometric contraction): between the rectus femoris and biceps femoris (RF–BF), vastus lateralis and biceps femoris (VL–BF), vastus medialis and biceps femoris (VM–BF), and lateral gastrocnemius and tibialis anterior (LG–TA), using the following equation [39,41]:

(3) $$\;CI=\frac{\left[\sum_{i=1}^{n}\frac{\mathrm{lower}\;\mathrm{EMGi}}{\mathrm{higher}\;\mathrm{EMGi}}\;\left(\mathrm{lower}\;\mathrm{EMGi}+\mathrm{higher}\;\mathrm{EMGi}\right)\right]}{n}$$

where i is the sample number and *n* is the number of data samples in the interval. Lower EMGi is the minimum value of the muscle during electromyography, while a higher EMGi is the maximum value of the muscle. This method does not take into consideration that a muscle may act as agonist or antagonist during the drop jump. In fact, in the formula: the numerator is represented by a lower EMG value and the denominator by the highest EMG value. This is important when analyzing drop jumps (i.e., in the aerial phase the muscle actions are indistinguishable). The main advantage of using this equation is that it provides a quick indication of the amount of simultaneous activations of muscles independently from their action (agonist–antagonist). This implies that, in a defined time interval (i.e., pre-contact phase), a pair of muscles can reverse their muscle activation (lower-numerator/higher denominator and vice versa), therefore, the calculation is performed by the summation of the different sample number (i) divided by the number of data samples in the interval (n).

The coactivation index is expressed as a percentage (%), and the maximum value is equal to 200% (meaning that both muscles were activated to their maximum value). In each participant, the CI index was calculated at the several drop heights in each phase of the drop jump (i.e., pre-contact, braking and propulsion phase) (Figure 1). The pre-contact phase was defined as the 0.10 s period prior ground contact [3].

#### 2.3.2. Power and Velocity

Velocity was obtained by integrating the ratio between the different forces (ground reaction force, *F_g_* and weight force, *F_w_*) and mass over time [40]:

(4) $$\int_{t_{1}}^{t_{2}}F\;dt=m\;(V_{2}-\;V_{1})$$

From the aforementioned equation, it is possible to determine the velocity:

(5) $$v=\;\frac{\int_{t_{1}\;}^{t_{2}}\left(\;F_{g}\;-\;F_{w}\;\right)dt\;}{m}$$

Instantaneous power (*P*) was calculated as the product of force (*F*) and velocity (*v*):

(6) Power = Force ×Velocity

### 2.4. Statistical Analysis

The data of males and females were pooled together because the subjects were recruited from a population where gender provided response differences in terms of absolute magnitude but not in terms of shape patterns in function of different drop heights [15,42,43].

The effect of the independent variable (drop height) on the dependent variable (ground reaction force peak, power, stiffness and coactivation index) was determined using a Friedman test, setting the type 1 error at 5%. The Bonferroni correction was used to adjust the *p*-values according to the number of comparisons that were performed, and post hoc analysis was performed to quantify the Cohen’s effect size (ES) for the dependent variables when the contrasts were significant. The intra-day reliability of the dependent variables was quantified by using the intra-class correlation coefficient (ICC, 95% confidence limit, lower confidence limit-upper confidence limit).

A linear mixed regression was used to model how leg stiffness and power propulsion were related to the coactivation of leg muscles involved in the different jump phases and different heights of drop jumps. Random intercepts (N(β_0_,σ_0_)) at an individual level and random slopes at the height level (N(β_1_,σ_1_)) were tested. Analysis was carried out using STATA statistical software (StataCorp LLC-Lakeway Drive, Texas, TX, USA, version 15).

## 3. Results

### 3.1. Measurements Reliability

The averaged intra-class correlation coefficient (ICC) of the sEMG_RMS_ values for each muscle and for ground reaction force were: 0.80, 0.83, 0.88, 0.74, 0.82, 0.83 and 0.89, respectively, for RF, VM, VL, BF, TA, LG and ground reaction force. In Table S1, all the values are reported.

The knee angle displacement among the several drop heights ranged from 121.8 ± 3.56 to 124.4 ± 3.55 degrees. However, the differences in knee angle displacement among the several trials performed at different drop heights were not significant (*p* > 0.05) and the ICC was equal to 0.89 (confidence interval 95%; the lower confidence limit was equal to 0.78; the upper confidence limit was equal to 0.96). The typical error as a CV was equal to 4.1% (lower limit 3.4%; upper limit 5.3%).

### 3.2. Ground Reaction Force, Power and Leg Stiffness

**Figure 2:**
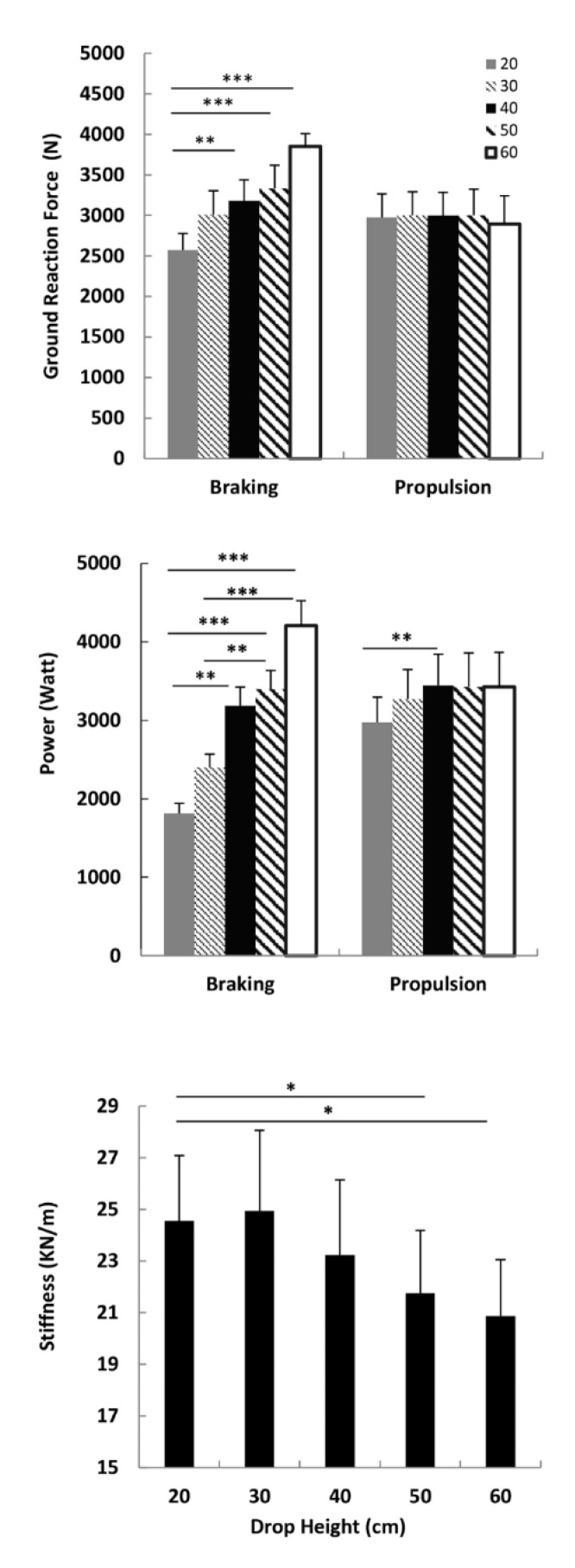
Mean values (SE) of the ground reaction force, power and leg stiffness reported during the drop jumps performed from five drop heights (20, 30, 40, 50 and 60 cm). * Statistical significance (* *p* < 0.05; ** *p* < 0.001; *** *p* <0.0001).

The ground reaction force increased significantly (*p* < 0.0001) as the drop height increased from 20 to 60 cm during the braking phase, and significant contrasts were found between 20 and 40 cm (*p* < 0.005; ES = 0.65), 20–50 cm (*p* < 0.0001; ES = 0.74) and between 20 and 60 (0.0001; ES = 1.34) (Figure 2). In contrast, no significant changes occurred during the propulsion phase (*p* > 0.05). Power increased significantly from 20 to 60 cm in the braking phase (*p* < 0.0001) (Figure 2). Significant contrasts were found between 20 and 40 cm (*p* < 0.0001; ES = 1.36), 20 and 50 cm (*p* < 0.0001; ES = 1.46), 20 and 60 cm (*p* < 0.0001; ES = 1.57), 30 and 50 cm (*p* < 0.01; ES = 1.1), and 30 and 60 cm (*p* < 0.0001; ES = 1.36) (Figure 2). The power in the propulsion phase increased from 20 to 50 cm (*p* < 0.05), with a significant difference between 20 and 40 cm (*p* < 0.01; ES = 0.34) (Figure 2). Conversely, the leg stiffness showed a significant decrease (*p* < 0.05) when the drop height increased from 20 to 60 cm, and significant contrasts were found between 20 and 50 cm (*p* < 0.05; ES = 0.25) and between 20 and 60 cm (*p* < 0.05; ES = 0.28) (Figure 2).

### 3.3. Coactivation Index and Drop Height

**Figure 3:**
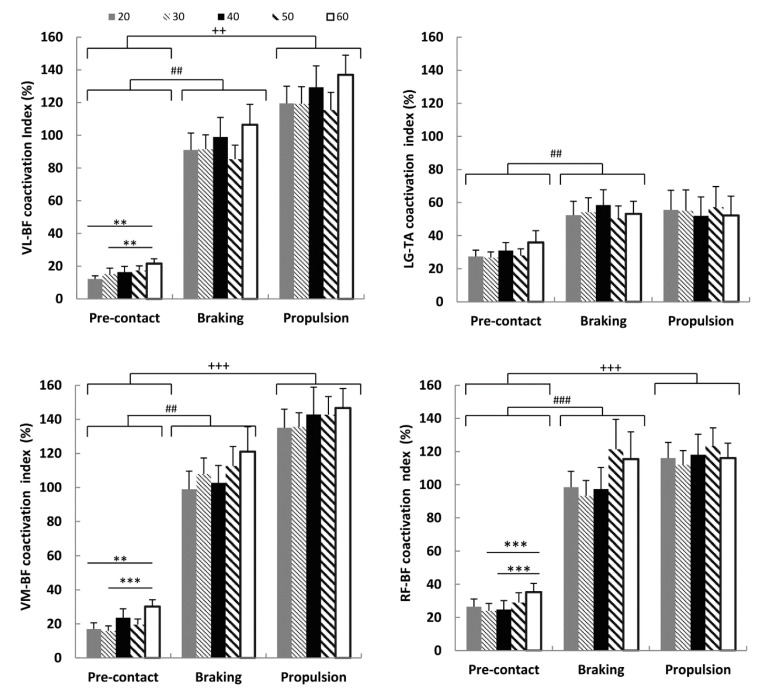
Mean values (SE) of the coactivation index are reported in three different phases (pre-contact, braking, propulsion) of the drop jump (DJ) and from five drop heights (20, 30, 40, 50 and 60 cm). The coactivation index is reported for the following pairs of muscles: vastus lateralis and biceps femoris (VL–BF), lateral gastrocnemius and tibialis anterior (LG–TA), vastus medialis and biceps femoris (VM–BF), rectus femoris and biceps femoris (RF–BF). * Statistical significance among the drop heights within each phase (** *p* < 0.01; *** *p* <0.0001). ^#^ Statistical significance between the pre-contact phase and braking phase (average values) (^##^ *p* < 0.01; ^###^ *p* < 0.0001). ^+^ Statistical significance between the pre-contact phase and propulsion phase (^++^ *p* < 0.01; ^+++^ *p* < 0.0001).

The VL–BF index showed a significant increase with increasing drop height in the pre-contact phase (*p* < 0.01). Significant differences were found between 20 and 60 cm (*p* < 0.01; ES = 0.89) and between 30 and 60 cm (*p* < 0.01; ES = 0.60). The VL–BF index did not significantly increase as a function of drop height during the braking phase or the propulsion phase (*p* > 0.05). The average VL–BF index of the propulsion phase was higher than the average value of the pre-contact phase (*p* < 0.01; ES = 1.75). Similarly, the average index (VL–BF) of the braking phase was significantly higher than that of the pre-contact phase (*p* < 0.01; ES = 2.00) (Figure 3).

The LG–TA index did not change significantly as a function of drop height during any phase (*p* > 0.05), whereas the average index of the braking phase was significantly higher than the average index of the pre-contact phase (*p* < 0.01; ES = 0.71) (Figure 3).

The VM–BF index of the pre-contact phase increased as a function of drop height (*p* < 0.01). The significant differences were located between 20 and 60 cm (*p* < 0.01; ES = 0.83) and between 30 and 60 cm (*p* < 0.0001; ES = 0.90). The VM–BF index did not significantly increase as a function of drop height during the braking phase or the propulsion phase (*p* > 0.05). The average VM–BF index of the braking phase was higher than that of the pre-contact phase (*p* < 0.01; ES = 1.70), and the average VM–BF index of the propulsion phase was higher than that of the pre-contact phase (*p* < 0.0001; ES = 1.70) (Figure 3). Similarly, the RF–BF index of the pre-contact phase increased as a function of drop height (*p* < 0.01), showing significant differences between 30 and 60 cm (*p* < 0.01; ES = 0.45) and between 40 and 60 cm (*p* < 0.01; ES = 0.57). The index of the braking and propulsion phases did not increase significantly as a function of drop height (*p* >0.05).

The average RF–BF index of the braking phase was greater than that of the pre-contact phase (*p* < 0.0001; 1.53), and the average RF–BF index of the propulsion phase was higher than that of the pre-contact phase (*p* < 0.0001; ES = 1.75) (Figure 3).

### 3.4. Coactivation Index, Power and Leg Stiffness

Leg stiffness was positively affected by the coactivation of VM–BF and RF–BF (*p* = 0.034; *p* = 0.046, respectively) (Table 1). In contrast, power propulsion was positively affected by the coactivation of LG–TA (*p* = 0.002) (Table 2).

## 4. Discussion

The objective of this study was to investigate the effects of drop heights on leg stiffness and power propulsion. Additionally, the coactivation strategies used by thigh and shank muscles in function of different drop heights and during each phase of the jump were studied. The main results, in accordance with our hypothesis, were (a) the drop heights modulated differently leg stiffness and power propulsion; (b) the coactivation of the thigh muscles was dependent on drop height only during the pre-contact phase and it increased during the braking and propulsion phases, although neither was dependent on drop height; (c) the coactivation of shank muscles was not dependent on drop height; and (d) the coactivation of thigh muscles influenced leg stiffness, whereas the shank muscle influenced the power propulsion.

Firstly, our results show that higher drop heights (higher momentum at impact) determine an increase in ground reaction force and power during the braking phase and a decrease in leg stiffness (Figure 1). This stiffness pattern seems to be conflicting with the coactivation index increase in the thigh muscles in function of drop height (VL–BF, VM–BF and RF–BF), during the pre-contact phase. From a functional point of view, the coactivation emphasizes that the feedforward control (or predictive control) plays a decisive role in the enhancement of muscle activity that was planned in advance and not altered by online peripheral feedback [5]. The anticipatory coactivation of the thigh muscles represents a protective strategy, necessary to withstand the increase in ground reaction forces that will occur at the impact when increasing the drop height, therefore, it stabilizes and prevents the knee joint collapsing immediately after the collision with the ground, allowing an efficient propulsion. This condition determined a constant amplitude of knee joint flexion (i.e., knee stiffness) on impact with the ground across drop heights [21].

The momentum of the body cannot be dampened by reflexes at the moment of impact, as these responses occur too late; in addition, the gain of the reflex might be too low to provide the required forces [1,17]. Therefore, the increase in muscle stiffness, which represents the only adjustable component by neural excitation, occurs prior to impact [18,19] through the coactivation of the knee muscles. The key factor in inducing a level of EMG amplitude appropriate to the drop height during the pre-contact phase is controlling the rate at which EMG activity during anticipation influences the timing and adjustment of motor responses [29,30]. In the present study, the coactivation increased significantly (VM–BF, VL–BF and RF–BF) after touch-down (i.e., the braking and propulsion phases) but it was not dependent on the drop height and this could indicate that the coactivation of thigh muscles is not sufficient to counteract the leg stiffness decrease that occurs at higher drop heights. However, the agonist–antagonist knee coactivation during dynamic contractions (i.e., drop jump) cannot be interpreted as an index of joint stabilization, the biceps femoris (BF) being a biarticular muscle and the moment of force produced antithetical to its anatomical classification and it is not necessarily produced in opposite direction to the movement [44].

This explanation is supported by a recent investigation by Helm et al. [29], which shows that RF and BF EMG activity are not in phase during the drop jump performed from 40 cm and under different experimental conditions (hard ground stiffness, soft ground stiffness, known and unknown the drop height); the RF muscle shows the highest medium latency response, while the BF muscle shows highest long latency responses.

The described motor strategies could be adopted to reduce joint stiffness in order to limit the risk of potential injuries at higher drop heights and/or favoring the capability of the muscle–tendon complex to absorb a greater amount of elastic energy [31]. In this regard, the trend to increase power propulsion from the drop height of 20 to 40 cm, observed in our result, could be due to the reuse of elastic energy since the ground reaction force remained constant (Figure 2).

Our results are not supported by one previous study [45], which reported that the coactivation of thigh muscles is not affected by drop height. This discordance in results could be explained by differences in biomechanical constraints. Specifically, in the present investigation, “drop jumps” were performed while maintaining an approximately constant knee angle at the different drop heights, as this is the condition necessary to develop higher power propulsion. This condition is probably responsible for the coactivation increase in the pre-programmed activity that prevents higher impact forces with higher drop heights from contributing to subsequent leg stiffness regulation and motor control [29]. Conversely, in the study by Kellis et al. [45], there was a decrease in the knee angle at higher drop heights, indicating that there was not necessarily an increase in the knee torque, since a reduction in knee angle flexion could have favored an increase in the moment arm [46]. Consequently, we could argue that the pre-programmed activity between the knee extensors and flexors in the study by Kellis et al. [45] did not change because the forces exerted around the knee were not altered.

Interestingly, our results showed that the coactivation of the shank muscles is not dependent on the drop height and they are supported by a previous investigation by Arai et al. [25] that observed a rebound height-dependent modulation of anticipatory coactivation of shank muscles; in other words, the highest rebound height was obtained by increasing the coactivation during the pre-contact phase.

The anticipatory coactivation has been also described during drop landings in normal and different conditions of simulated gravity [1,16,17,47,48,49]. The motor control during the drop landing is finalized to dissipate, after the touch down, the mechanical energy accumulated during the falling phase. When landing from a box without rebounding, the leg muscles change their mechanical properties from a spring to a damper [50]. The greater amount of energy is dissipated by an eccentric contraction of leg muscles and by the deformation of the soft tissues; anyway, the motor control strategy is involved to regulate the magnitude of the collision and the amount of negative work. Therefore, the landing strategy can be stiff (greater impact force) or soft (greater negative work) depending on the ratio between the ground rection force and the negative mechanical work performed by the joints. In this regard, the anticipatory coactivation is responsible for increasing the joints stiffness necessary to increase the stability at the impact.

The neural strategy during a drop landing is organized to increase ankle stiffness depending on the drop height by means of the coactivation of the soleus and tibialis anterior muscles before and after contact with the ground. Indeed, the increase in magnitude of the ground reaction force with the drop height is not accompanied by a similar increase in ankle rotation [17]. This implies that the muscles around the knee and hip joints show a larger range of motion and contribute to the absorption of the landing impact and dissipate the energy [16]. In contrast to the drop landing; the energy accumulated in the aerial phase of the drop jump could be reused in the subsequent push-off phase to increase the power propulsion when the stretch–shorten cycle is performed with specific biomechanical constraints. We found that the coactivation of shank muscles during the braking phase optimized power propulsion, while the coactivation of thigh muscles provided optimal stiffness to regulate the deceleration of the joint at ground impact (Table 1 and Table 2). In other words, by increasing the drop height, the role of the leg joints in the two drop conditions changes on impact with the ground. To be precise, in the drop landing condition, the ankle joint does not perform significant angular displacement while the knee rotates, and the thigh muscles that are involved absorb the kinetic energy [16]. On the other hand, in the drop jump condition (bounce drop jump), the knee joint shows less angular displacement; thus, the increase in kinetic energy may be stored more easily in the muscle–tendon complex as the ankle joint is less stiff [31].

Our interpretation is in agreement with two studies carried out by Lesinski et al. [51,52]. In the first study [51], by increasing the drop heights, the anticipatory muscle activity of shank muscles increased progressively while the anticipatory coactivation of the same muscles decreased; this condition could favor the elastic energy storage more efficiently during the ground contact to enhance jump performance. Moreover, the knee angle flexion also increased in function of drop height and the maximum was observed at 60 cm, probably to dampen the higher impact load. The authors argued that the subjects manipulate the knee angle to adjust leg/knee joint stiffness [52].

However, in comparing our results with those reported in the literature, the differences in the experimental design must be taken into account (i.e., equation used to calculate the coactivation index, type of jump).

### Limitation

In the literature, gender-specific effects on kinetic, kinematic and motor control strategies during drop jump have been documented [15,42,53,54]. Recently, these differences have been attributed to a different feedforward control strategy; females show a deficit in the rate tension developed by hip extensors and consequently they have to activate the knee extensor earlier than males to counteract their deficit [55]. Therefore, we are aware that gender is a confounder affecting magnitude responses, however, as the main goal of the present study is detecting the shape response in function of different drop heights [15,43,52], we decided to drop this variable to prevent power issues regarding the overall neuromuscular pattern responses.

Concerning the recruitment of participants, it was limited to sport science students and not to experienced jumpers because the former may increase the generalizability of the results considering that drop jump exercises are not only performed to improve the reactive strength of athletes but also to prevent interventions [24,56,57]. This is the reason why our study should be generalized with caution to athletes. 

## 5. Conclusions

In summary, coactivation was dependent on drop height only during the pre-contact phase and mainly involved the thigh muscles. Additionally, the coactivation of the knee extensors and flexors during the braking phase (VL–BF and VM–BF) explains leg stiffness adjustments and motor control. The coactivation of ankle extensors and flexors (LG–TA) during the braking phase is responsible for the power propulsion increase, probably by optimizing the storage and recoil of elastic energy in the triceps surae muscle.

These results underline the mismatch of the drop heights to maximize leg stiffness and power propulsion. The perspective for future longitudinal studies could indicate drop heights of 20–30 cm to increase the functional role of co-contraction in joint stability and injury protection, considering that leg stiffness is at its highest, whereas higher drop heights should be considered to optimize power propulsion in power training interventions.

## Figures and Tables

**Table 1:** Leg stiffness linear mixed model coactivation estimates.

Leg Stiffness	$\hat{\beta }$	SEM	*p* Values
Drop height	−1.487	0.486	0.002
VM–BF	0.054	0.025	0.034
VL–BF	0.043	0.028	0.127
RF–BF	0.045	0.022	0.046
LG–TA	0.002	0.042	0.961

Coefficient of regression: β, standard error of measurement: SEM, rectus femoris: RF, vastus medialis: VM, biceps femoris: BF.

**Table 2:** Power propulsion linear mixed model coactivation estimate.

Power Propulsion	$\hat{\beta }$	SEM	*p* Values
Drop height	124.8	42.22	0.003
VM–BF	2.820	2.387	0.238
VL–BF	2.573	2.690	0.339
RF–BF	3.259	1.990	0.101
LG–TA	17.74	5.786	0.002

Coefficient of regression: β, standard error of measurement: SEM, lateral gastrocnemius: LG, tibialis anterior: TA.

## Supplementary Materials

The following are available online at https://www.mdpi.com/1660-4601/17/22/8647/s1, Table S1: Reliability intra-session of the measured variables during the different drop heights and phases of the drop jumps.
