# Supplementary material for: Neuromuscular Strategies in Stretch–Shortening Exercises with Increasing Drop Heights: The Role of Muscle Coactivation in Leg Stiffness and Power Propulsion
Source: Int J Environ Res Public Health. 2020 Nov 21;17(22):8647. doi: 10.3390/ijerph17228647 (PMC7700220; doi:10.3390/ijerph17228647)
Supplement: Supplementary file 1 [file ijerph-17-08647-s001.pdf]

Table S1. Reliability intra-session of the measured variables during the different drop heights and phases of the drop jumps.

	PHASE	RF	VM	VL	BF	TA	LG	GRF
**DJ 20**	**Pre-contact**	0.75	0.80	0.78	0.66	0.84	0.76	
	**Breaking**	0.77	0.81	0.91	0.55	0.86	0.91	0.91
	**Propulsion**	0.89	0.88	0.96	0.85	0.89	0.89	0.99
**DJ 30**	**Pre-contact**	0.84	0.88	0.66	0.83	0.72	0.74	
	**Breaking**	0.72	0.85	0.91	0.81	0.79	0.78	0.74
	**Propulsion**	0.83	0.92	0.95	0.81	0.81	0.93	0.96
**DJ 40**	**Pre-contact**	0.89	0.87	0.86	0.78	0.63	0.67	
	**Breaking**	0.80	0.95	0.92	0.78	0.88	0.97	0.80
	**Propulsion**	0.59	0.90	0.96	0.87	0.86	0.98	0.99
**DJ 50**	**Pre-contact**	0.87	0.66	0.86	0.81	0.87	0.89	
	**Breaking**	0.80	0.93	0.93	0.49	0.82	0.94	0.93
	**Propulsion**	0.84	0.97	0.94	0.57	0.96	0.98	0.95
**DJ60**	**Pre-contact**	0.68	0.82	0.89	0.76	0.76	0.88	
	**Breaking**	0.89	0.63	0.83	0.83	0.67	0.42	0.70
	**Propulsion**	0.78	0.62	0.90	0.67	0.87	0.65	0.89
**Mean**		0.80	0.83	0.88	0.74	0.82	0.83	0.89

Abbreviations: rectus femoris (RF), vastus medialis (VM), vastus lateralis (VL), biceps femoris (BF), tibialis anterior (TA), lateral gastrocnemius (LG), and ground reaction force (GRF).
